# The effects of repeated doses of xylazine-ketamine and medetomidineketamine anesthesia on DNA damage in the liver and kidney

**DOI:** 10.1590/acb385723

**Published:** 2023-10-30

**Authors:** Tunahan Sancak

**Affiliations:** 1 Sivas Cumhuriyet University – Veterinary Faculty – Department of Surgery – Sivas – Turkey.

**Keywords:** Anesthesia, DNA Damage, Gene Expression, Repeated Dose, Tumor Necrosis Factor-alpha

## Abstract

**Purpose:**

This study evaluated the DNA damage caused by repeated doses of xylazine-ketamine and medetomidine-ketamine anesthesia in the liver and kidneys.

**Methods:**

In this study, 60 rats were used. The rats were divided into group 1 (xylazine-ketamine), and group 2 (medetomidine-ketamine), and these anesthetic combinations were administered to the rats at repeated doses with 30-min intervals. The effects of these anesthetic agents on the tumor necrosis factor-alpha gene for DNA damage were investigated.

**Results:**

According to the gene expression results, it was observed that a single dose of xylazine-ketamine was 2.9-fold expressed, while first and second repeat doses did not show significant changes in expression levels. However, in the case of the third repetition, it was observed to be 3.8-fold overexpressed. In the case of medetomidine-ketamine administration, it was observed that a single-dose application resulted in a 1.04-fold expression, while the first and the third repeat doses showed a significant down expression. The samples from the second repeat dose administration group were found to have insignificant levels of expression.

**Conclusions:**

This study can contribute to understanding the safe anesthetic combination in research and operations in which xylazine-ketamine and medetomidine-ketamine combinations are used.

## Introduction

Alpha2 adrenoreceptor agonists (xylazine, medetomidine, etc.) have a number of advantages over other drugs when used as a premedication for general anesthesia. These include sedation, anxiolysis, analgesia, inhibition of autonomic reflex response, decreased anesthetic needs, enhanced intraoperative stability, and facilitation of anesthesia induction. General anesthetics are agents that can produce reversible unconsciousness, amnesia, analgesia (absence of pain), muscle relaxation and immobility^1^^,^ 2. The combination of medetomidine/ketamine and xylazine/ketamine is described as a useful anesthesia technique frequently used in surgical procedures in animals^3^^,^ 4. They quickly absorb and are evenly disseminated throughout the central nervous system after injection. Xylazine, medetomidine, and ketamine are metabolized by the liver and excreted mainly by the kidney. Therefore, their use is contraindicated in animals with dysfunction of the liver and kidneys^5^^-^ 7.

Studies have reported that the half-lives of xylazine and medetomidine are approximately 30 minutes, and the plasma half-life of ketamine is between 27–68 minutes, depending on the dose^8^^-^ 10.

In case the surgical procedure cannot be completed during the initial phase of anesthesia in animals, maintenance doses of anesthetic agents such as medetomidine, xylazine, and ketamine are used^11^^-^ 14. With repeated usage, these agents can have modest adverse effects, such as increased anxiety-related behaviors, although they are generally well tolerated^15^.

This study evaluated the DNA damage caused by repeated doses of xylazine-ketamine and medetomidine-ketamine anesthesia in the liver and kidneys.

## Methods

According to the decision of the Sivas Cumhuriyet University Local Ethics Committee for Animal Experiments, dated October 6, 2022, with the reference number 65202830-050.04.04-674, this study was carried out.

A total of 60 male Wistar albino rats, weighing between 200 and 250 g on average, have been used in the research. The animals were fed freely and kept in rooms with averaged 26°C and 60% relative humidity, with a 12-h light-dark cycle.

### Animal experiments

Sixty rats were used for the study, divided into two main groups:

- Group 1: xylazine-ketamine group;
- Group 2: medetomidine-ketamine group.

Furthermore, these groups were further divided into subgroups based on dosage frequency.

For anesthesia, group 1 (xylazine-ketamine group) received xylazine hydrochloride (Xylazinbio, Bioveta, Czech Republic) at the dose of 3 mg/kg and ketamine hydrochloride (Ketasol, Richter Pharma, İnterhas, Ankara) at the dose of 90 mg/kg. Group 2 (medetomidine-ketamine group) received medetomidine (Domitor, Orion Pharma, Zoetis, Istanbul) at the dose of 0.15 mg/kg and ketamine hydrochloride at the dose of 90 mg/kg. Additionally, repeated doses were administered at the level of ½ of the first dose.

The control group was formed to take samples from the liver and kidney based on the half-lives of xylazine, medetomidine and ketamine. Control group samples were used for both normalization of the expression levels in the groups and standardization of the expression level that should be at a normal level and to determine the difference.

In group 1 (xylazine-ketamine group), 30 rats were induced into general anesthesia by administering the combination of xylazine and ketamine. The procedures performed on the rats were as follows:

- Control Group: this group consisted of randomly selected six rats. The combination of xylazine and ketamine was administered to these six rats to induce general anesthesia. As soon as the rats lost reflex responses (1.6–6.3 min^16^), they were immediately sacrificed.
- Single-dose administration: six rats were randomly selected from the remaining 24 rats and received a single dose of xylazine-ketamine combination. After 30 min of single-dose administration, while the rats were under anesthesia, exsanguination was performed to sacrifice them.
- First repeat dose: six rats were randomly selected from the remaining 18 rats and received two consecutive doses of the xylazine-ketamine combination with a 30-min interval between doses. After 30 min from the second administration, while the rats were under anesthesia, exsanguination was performed to sacrifice them;
- Second repeat dose: six rats were randomly selected from the remaining 12 rats and received three consecutive doses of the xylazine-ketamine combination with a 30-min interval between each dose. After 30 min from the third administration, while the rats were under anesthesia, exsanguination was performed to sacrifice them;
- Third repeat dose: the remaining six rats, after random selection from the other groups, received four consecutive doses of the xylazine-ketamine combination with a 30-min interval between each dose. After 30 min from the fourth administration, while the rats were under anesthesia, exsanguination was performed to sacrifice them.

In group 2 (medetomidine-ketamine group), 30 rats were subjected to medetomidine-ketamine administration. Similar to the rats in the group 1, the rats in this group were divided into a control group and four subgroups, and the same procedures were performed using the medetomidine-ketamine combination.

After sacrificing all the rats, liver and kidney tissues were collected and stored under appropriate conditions for further analysis. The samples were sent to the laboratory for the relevant analyses.

### RNA isolation

A total of 25 mg of tissue was collected from all experimental group samples, and isolation was performed using the RNeasy Mini Kit (Qiagen) on the QIAcube device. The protocol of the QIAcube device was optimized for the elution step of RNA isolation. To homogenize the tissue, 350 μL of RTL buffer was added to the 25-mg tissue and homogenized using the Tissue Lyser LT (Qiagen). The homogenate was transferred to a 2-mL Eppendorf tube, and 600 μL of 70% ethanol was added and pipetted. Centrifugation was performed at 8,000 g for 15 sec. Next, 700 μL of RW1 buffer was added, and centrifugation was performed at 8,000 g for 15 sec. Then, 500-L of RPE buffer was added, and the spin column was centrifuged at 8,000 g for 15 sec after being moved to a new collecting tube. So, 500-L of buffer was added, the RPE spin column was moved to a new collecting tube, and the mixture was centrifuged at 8,000 g for 2 min. Finally, 30-L of RNase-free water was added, and RNA elution was obtained by centrifuging at 8,000 g for 1 min^17^.

### cDNA synthesis

All steps of cDNA synthesis were performed on a cold plate at +4 °C. The RT2 First Strand cDNA synthesis kit (Qiagen) was used for cDNA synthesis. The isolated 1 μL RNA was equalized to a concentration of 100 ng and brought up to a total volume of 10 μL. Then, 2 μL of GE buffer was added to the total volume, resulting in a final volume of 12 μL. Subsequently, it was incubated in a thermal cycler device at 42 °C for 5 min. At the end of the incubation period, 5X reaction buffer (4 μL), primer (2 μL), and reverse transcriptase mix (2 μL) were added to complete the final volume of 20 μL. The final volume was then incubated at 42 °C for 15 min, followed by incubation at 95 °C for 5 min in a thermal cycler device^18^.

### Gene expression analysis

The cDNA products were subjected to real-time polymerase chain reaction (PCR) analysis using the RotorGene Q 9000 device (Qiagen) and RT2 SYBRGreen quantitative PCR (qPCR) master mix (Qiagen). The β-actin gene was chosen as the reference gene for gene expression analysis. The tumor necrosis factor (TNF)-α gene was selected as the target gene, and its sequence data were obtained from the NCBI gene bank to design the primers. The primers used in the study and their specifications are provided in Table 1.

**Table 1:** Primers used in the study and their properties^4^^,^ 19.

Gene	Sequence F	bp	Sequence R	bp	tm
β-actin	AGG GAA ATC GTG CGT GAC	18	GGA AGA GAG CCT CGG GG	18	55
TNF-α	TCC TTC CTG ATC GTG GCA	18	TGA AGA GGA CCT GGG AGT AGA T	22	55

Source: Wang et al.^4^ and Bonefeld et al.^19^.

PCR mixture was prepared by adding 12.5 μL of SYBRGreen qPCR master mix (Qiagen), 1 μL of forward and reverse primer each, and 5.5 μL of H_2_O, bringing the total volume to 20 μL. Then, 5 μL of cDNA was added to the total volume, resulting in a final volume of 25 μL. The real-time PCR protocol was completed with cycling conditions consisting of denaturation at 95°C for 30 sec, followed by 45 cycles of 5 sec at 95°C and 30 sec at 55°C^20^.

### Statistical analysis

After PCR analysis, gene expression levels were determined using 2ΔΔct -log values based on the ct values. Normalized values were analyzed using one-way analysis of variance (ANOVA) to determine the significance of differences between mean values. Duncan’s multiple test and paired data differences test were used to assess the significance of differences between mean values. Statistical significance was set at p < 0.05^21^.

## Results

### Gene expression analysis

**Figure 1:**
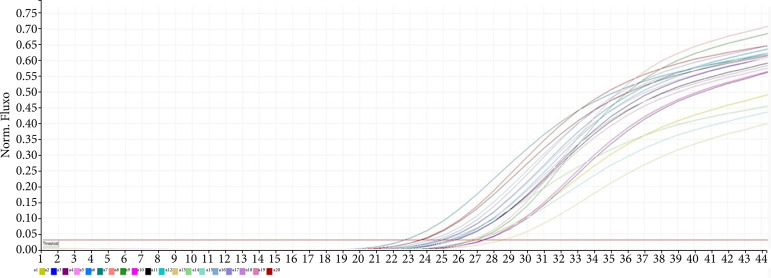
β-actin gene real-time polymerase chain reaction result.

Source: Elaborated by the author.

In the study, the β-actin gene was used as a housekeeping gene in the real-time PCR analysis. It was observed that the automatic threshold value set by the device was 0.03. It was found that all samples had ct values above the threshold value. The gene expression values of the TNF-α gene were determined using the ct values of the housekeeping gene. The real-time PCR result for the β-actin gene is shown in Fig. 1.

**Figure 2:**
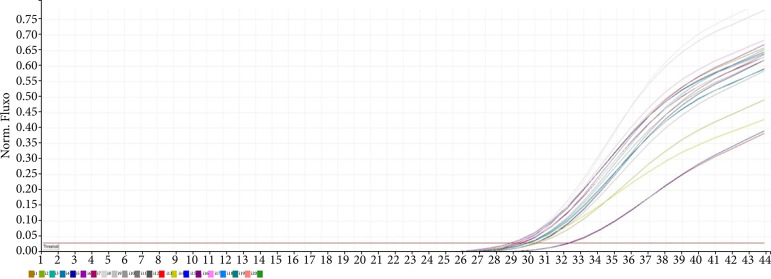
Real-time polymerase chain reaction result of tumor necrosis factor-α gene.

Source: Elaborated by the author.

Figure 2 shows the graph representing the TNF-α gene expression levels obtained from the normalization and formulation of the ct values obtained from the real-time PCR results. The graph displays the fold values and 2ΔΔct -log values, representing the TNF-α gene expression levels.

**Figure 3:**
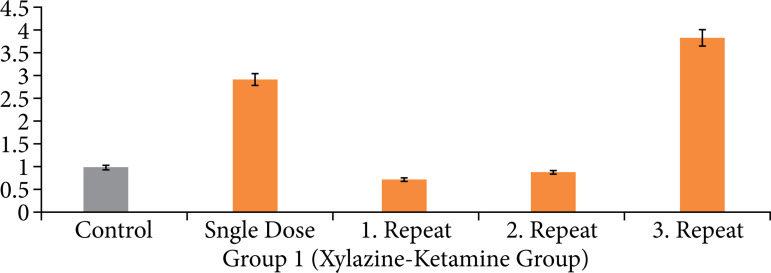
Tumor necrosis factor-α gene expression levels observed from the gene expression analysis for group 1 (xylazine-ketamine group).

Source: Elaborated by the author.

In accordance with the statistical results of the ct values obtained after PCR analysis, the expression level differences in line with the application differences obtained for the xylazine-ketamine group are given in Fig. 3.

According to the TNF-α gene expression results of the xylazine-ketamine group, it was observed that the expression level of the control group was normalized to 1. Based on this value, it was found that the single-dose administration resulted in a 2.9-fold increase in expression. The first and second repeat-dose administrations showed no significant changes in expression levels, while the third repeat-dose administration resulted in a 3.8-fold overexpression.

**Figure 4:**
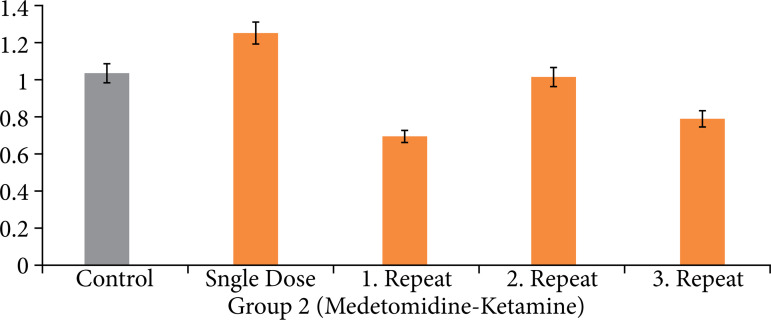
Tumor necrosis factor-α gene expression levels observed from the gene expression analysis for group 2 (medetomidine-ketamine group).

Source: Elaborated by the author.

Regarding the medetomidine-ketamine group, the expression level differences based on the variations in administration were depicted in Fig. 4, considering the statistical results of the ct values obtained from the PCR analysis.

According to the TNF-α gene expression results of the medetomidine-ketamine group, it was observed that the expression level of the control group was normalized to 1. Based on this value, it was found that the single-dose administration resulted in a 1.04-fold increase in expression. First and third repeat-dose administrations showed significant downregulation in expression levels, while the second repeat-dose administration group exhibited insignificant changes in expression.

**Figure 5:**
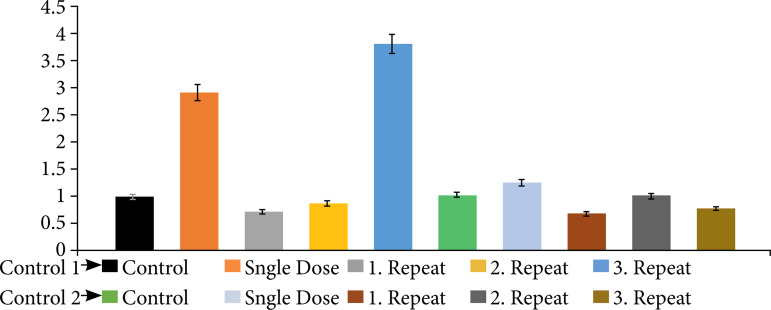
Tumor necrosis factor-α gene expression levels observed from the gene expression analysis for group 1 (xylazine-ketamine) and group 2 (medetomidine-ketamine).

Source: Elaborated by the author.

A graph illustrating the expression levels that can be observed for both administrations and all groups together is provided in Fig. 5.

## Discussion

Xylazine, medetomidine and ketamine have an average half-life of approximately 30 min^9^^,^ 10 ^,^ 22. A study conducted by Gehring et al.^23^ determined the half-life of ketamine in xylazine-ketamine anesthesia to be approximately 30 min. Xylazine-ketamine or medetomidine-ketamine mixtures provide anesthesia for approximately 30 min. Half of the initial dose of anesthetic mixture can be repeated as additional dose if necessary (insufficient sedation, surgical procedure cannot be completed, etc.)^11^^,^ 24. In light of this information, in this study, xylazine-ketamine and medetomidine-ketamine mixtures were administered as maintenance doses at 30-min intervals after the initial application, and, after these periods, tissue samples were taken from the liver and kidneys after sacrificing the rats from each group.

Acute liver and kidney injury are primarily caused by inflammation, and systemic inflammatory responses are key players in the pathophysiology of these injuries. During inflammation, TNF-α is one of the major pro-inflammatory enzymes that regulate both innate and adaptive immune responses, and high levels of DNA damage increase TNF-α production, leading to the activation of signaling molecules such as NF-κB. TNF-α mRNA expression plays a significant role in liver and kidney damage^4^^,^ 25 ^-^ 27.

It has been reported that the induction of general anesthesia increases the plasma concentration of pro-inflammatory cytokine TNF-α and induces the release of TNF-α from blood cells^28^. Mastronardi et al.^29^ observed an increase in TNF-α levels following the administration of ketamine/acepromazine/xylazine. Rocha et al.^30^ investigated the effects of sevoflurane and isoflurane on DNA damage in rats and demonstrated that sevoflurane caused higher levels of DNA damage compared to isoflurane. Leffa et al.^31^ showed that xylazine-ketamine administration resulted in DNA damage in the liver and kidneys of rats. Došenović et al.^32^ conducted a study on turtles and found that medetomidine-ketamine anesthesia did not cause DNA damage. Similarly, in this study, it was determined that xylazine-ketamine administration caused higher levels of DNA damage in the liver and kidneys compared to medetomidine-ketamine administration (Fig. 5).

According to the gene expression results, it was observed that single-dose applications of xylazine and medetomidine significantly increased the expression levels of genes associated with DNA damage (Figs. 1 and 2). Subsequent first and second repeat dose administrations did not influence the extent of DNA damage, but the third repeat dose of xylazine reactivated DNA damage (Figs. 3 and 5). In the case of medetomidine administration, it was observed that, after the initial application’s damaging effect on DNA, the subsequent administrations caused lower levels of damage or did not induce damage again after the initial damage (Figs. 4 and 5).

Based on these findings, it can be said that anesthetic substances exhibit their highest level of effect in the body during the initial applications, but in some anesthetic substances the rate of damage increases with the number of administrations.

## Conclusion

In conclusion, both single and repeated dose administrations have shown that medetomidine-ketamine induces less expression of TNF-α and, consequently, causes less DNA damage in the liver and kidneys of rats compared to the xylazine-ketamine combination. It is therefore considered that the medetomidine-ketamine combination may be safer than the xylazine-ketamine combination in long-term operations in which repeated doses may be necessary.

## Data Availability

All data sets were generated or analyzed in the current study.
